# Antimicrobial and Antiviral Compounds of *Phlomis viscosa* Poiret

**DOI:** 10.3390/biomedicines11020441

**Published:** 2023-02-02

**Authors:** Ludmila Yarmolinsky, Faina Nakonechny, Arie Budovsky, Haim Zeigerman, Boris Khalfin, Eyal Sharon, Leonid Yarmolinsky, Shimon Ben-Shabat, Marina Nisnevitch

**Affiliations:** 1 Eastern R&D Center, Kiryat Arba 9010000, Israel; 2 Department of Chemical Engineering, Ariel University, Ariel 4070000, Israel; 3 Research & Development Authority, Barzilai University Medical Center, Ashkelon 7830604, Israel; 4 Institute of Biochemistry, Food Science and Nutrition, The Robert H. Smith Faculty of Agriculture, Food and Environment, Hebrew University of Jerusalem, P.O. Box 12, Rehovot 7610001, Israel; 5 Faculty of Health Sciences, Ben-Gurion University of the Negev, Beer-Sheva 8410501, Israel; 6 Institute for Personalized and Translational Medicine, Ariel University, Ariel 4070000, Israel; 7 Arnie Miller Laboratories, Beer-Sheva 8430713, Israel

**Keywords:** *Phlomis viscosa*, antimicrobial compounds, antiviral compounds, flavonoids, drug-resistant microorganisms, biofilm formation

## Abstract

*Phlomis viscosa* Poiret (an evergreen shrub) represents a valuable source of medicinal compounds. In this study, we discovered compounds with antimicrobial and antiviral properties. The aim of this study was to identify compounds of *P. viscosa* and estimate the antimicrobial and antiviral activity of its phytochemicals. The volatile compounds were identified using gas chromatography/mass spectrometry (GC/MS) analysis. For the identification of nonvolatile components of the extracts, high-performance liquid chromatography (HPLC), liquid chromatography–electrospray ionization-mass spectrometry (LC-ESI-MS) and matrix-assisted laser desorption/ionization-time-of-flight mass spectrometry (MALDI-TOF-MS) were applied. Quercetin 3-*O*-rutinoside and hesperidin caused a significant decrease in the bacterial concentration of *Agrobacterium tumefaciens*, *Xylella fastidiosa* and *Pseudomonas syringae* (*p* < 0.001). The growth of drug-resistant microorganisms (*Escherichia coli*, *Klebsiella pneumoniae*, *Acinetobacter baumannii*, *Serratia marcescens* and *Salmonella enteritidis*) was inhibited by quercetin 3-*O*-rutinoside, quercetin 3-*O*-arabinoside and hesperidin. In addition, these compounds demonstrated antiquorum-sensing properties. Diosmin, hesperidin and quercetin 3-*O*-arabinoside significantly inhibited varicella zoster virus (VZV) (*p* < 0.001). Quercetin 3-*O*-rutinoside and quercetin 3-*O*-arabinoside were effective against herpes simplex virus 1 (HSV-1), including mutant strains.

## 1. Introduction

Genus *Phlomis* comprises more than 100 species of herbaceous plants, subshrubs and shrubs in the family *Lamiaceae*, native to Asia, southern Europe and northern Africa [1,2], but those of *P. viscosa* were not investigated in depth before 2019. *Phlomis viscosa* Poiret is an evergreen shrub that is native to Israel, Turkey, Lebanon and Syria [3,4]. This plant is a rich source of bioactive compounds with numerous therapeutic properties, including anti-inflammatory, antidiabetic, anticancer, pro-wound-healing and antimicrobial activities [4]. Traditional medicine uses *P. viscosa* in anti-inflammatory and dermatological remedies [1,2].

During recent decades, the unjustified overuse of antibiotics has provided selective pressure that has led to the widespread appearance of antibiotic-resistant microorganisms [5]. The acknowledgment that antibiotic resistance is one of the most important problems of modern medicine stresses the need to develop new approaches to fight these pathogens. One of them is the use of plant phytochemicals [6]. Medicinal plants and their active compounds may be used against drug-resistant microorganisms; however, the experimental data are not as complete as they could be for the development of new drugs [7].

Additionally, plant pathogens have garnered much interest as potential models for research since various plant diseases remain one of the most important agricultural problems worldwide [8]. For example, grapevines are often affected by the following bacterial diseases: crown gall caused by *Agrobacterium tumefaciens* [9], Pierce’s disease (*Xylella fastidiosa*) [10] and bacterial inflorescence rot (*Pseudomonas syringae*) [11]. The modern approaches to bacterial control are carried out by means of effective sanitation, robust diagnosis, local and regional certification efforts, antibiotics and the production of resistant grapevines [10,11]. These activities are extremely costly, and they ensure only partial success [12].

Another factor that makes many microorganisms more dangerous by enhancing genetic competence, bacterial colonization, biofilm formation and virulence is bacterial quorum sensing (QS)—the ability to detect and respond to cell population density by gene regulation [13]. Although some antimicrobial phytochemicals are also anti-QS compounds according to the bioassay-guided isolation [6,14], they have not been investigated deeply because of QS regulatory complexity.

Nine different types of herpesviruses infect humans and cause disease [15]. The primary infection is productive and results in the generation of infectious progeny virus. The infection eventually subsides and enters a latency phase, with restricted gene expression. Occasionally, the latent dormant virus is reactivated, again producing an infectious progeny virus. This pattern of infection imposes difficulties on drug design [16].

Currently, there are a limited number of antiherpetic drugs [17,18], and therefore, it is necessary to develop new antiviral agents.

Antiherpetic activities of some plant phytochemicals were reported earlier [19,20]. For example, mechanisms of polysaccharides’ efficiency against HSV include inhibiting either the absorption or penetration of HSV, direct killing of the HSV, inhibiting HSV biosynthesis and proliferation and activation of the immune system of the host [7].

It is widely acknowledged that the two most common causative agents of infectious disease are the virus and bacterium; viruses cannot be treated with antibiotics, nor bacteria with antivirals. The crude extract of *P. viscosa* was effective both against viruses and bacteria [4], and the question is which compounds are responsible for the antimicrobial and antiviral properties of this extract.

The anti-inflammatory, antidiabetic, anticancer [3] and antimicrobial [4] properties of *P. viscosa* were described earlier by us. It was found that the plant extract and a combination of some compounds enhance wound healing [4]. Additionally, in the studies of others, it was demonstrated that phenylethanoid glycosides isolated from *Phlomis viscosa* (Lamiaceae) exhibit weak antitumoral, antibacterial and antifungal activity [21]. The activity of these glycosides was tested towards Gram-positive bacteria *Staphylococcus aureus* and *Enterococcus faecalis*, Gram-negative *Escherichia coli* and *Pseudomonas aeruginosa* and three yeast-like fungi: *Candida albicans*, *C. krusei* and *C. parapsilosis*. The same compounds were tested versus three tumor cell lines SF-268, NCI-H460 and MCF7 [21].

In addition, an extract from *Phlomis viscosa* was found effective in inhibiting some phytopathogenic fungi [22].

To the best of our knowledge, individual components of *P. viscosa* extract were never tested against antibiotic-resistant bacteria and viruses.

The aim of this study was to identify individual components of *P. viscosa* extract and to study their activity against plant pathogens, drug-resistant microorganisms, biofilm formation and herpesviruses.

## 2. Materials and Methods

### 2.1. Preparation of Plant Material

Leaves and flowers of *P. viscosa* were ground and subjected to extraction. The plant material was treated as described previously [3,4,23].

### 2.2. Identification of Plant Extract Components

Identification of volatile compounds was performed by gas chromatography/mass spectrometry (GC/MS) analysis as described previously [3,4,23]. For the identification of nonvolatile components of the extracts, high-performance liquid chromatography (HPLC), liquid chromatography–electrospray ionization-mass spectrometry (LC-ESI-MS) and matrix-assisted laser desorption/ionization-time-of-flight mass spectrometry (MALDI-TOF-MS) were applied. For this purpose, an LC-MS Agilent 1100LC series (Waldbronn, Germany) and Bruker Esquire 3000plus MS (Bremen, Germany) instrument supplied by a C18 column (Betasil C18, 5 µm, 250 × 4.6 mm; Thermo Hypersil, Paisley, Scotland) and methanol-water (Bio-Lab Ltd., Jerusalem, Israel) (90:10 *v*/*v*) as the mobile phase were used. The UV detector was set at 360 nm, the flow rate was 1 mL/min and the injection volume was 10 µL. The MS conditions were as follows: API electron spray interface, negative mode polarity, a drying gas flow of 10 L/min, a nebulizer gas pressure of 60 psi, a drying gas temperature of 335 °C, a fragmentor voltage of 0.4 V and a capillary voltage of 4451 V. High-resolution MS. analysis was performed using a Bruker Reflex IV time-of-flight (TOF) mass spectrometer (Bruker Daltonics, Bremen, Germany). Ions were formed with the help of a pulsed nitrogen laser at 337 nm. Mass spectra were gained from an average of up to 350 single-shot spectra, the acceleration voltage was 25 kV and the delay was 150 ns.

### 2.3. Materials and Bacterial Strains

Lyophilized powders of *Agrobacterium tumefaciens* (ATCC 4720), *Xylella fastidiosa* (ATCC 2683), *Pseudomonas syringae* (ATCC 19310), *Escherichia coli* (ATCC 25922), *Klebsiella pneumoniae* (ATCC 43816), *Acinetobacter baumannii* (ATCC BAA1605), *Serratia marcescens* (ATCC 13880) and *Salmonella enteritidis* (ATCC 13076) were obtained from ATCC collection and recultivated using standard buffered peptone water (Acumedia™ 7365A, Lansing, MI, USA) according to the manufacturer’s instructions. Drug-resistant microorganisms were obtained from the laboratory at Soroka University Medical Center, Beer-Sheva, Israel. These bacteria were inoculated onto brain–heart infusion agar (BHA, Acumedia, Lansing, MI, USA).

Colonies of the bacteria were reinoculated into peptone water growing media and incubated at 35 °C for 24 h. After cultivation, the cultures were rinsed in phosphate buffered saline (PBS) (Acumedia, Neogen Corporation, Lansing, MI, USA) by centrifugation (6000 rpm) (Beckman Coulter, Brea, CA, USA) and resuspended in PBS (5 mL for each type of bacteria). The microbes were enumerated at time points of 0, 5 and 20 h by the heterotrophic plate count (HPC) method with pour plate inoculation onto selective agar media [4].

### 2.4. Biofilm Formation Estimation

For the crystal violet assay, 96-well polystyrene microplates were used [24]. The bacteria were grown for 48 h at 37 °C with or without the tested compounds or streptomycin. The nonadherent cells were washed out with sterile PBS, and adherent bacteria were stained for 10 min using a 1% crystal violet solution (Merck, Darmstadt, Germany).

### 2.5. Animal Cells and Viruses

Vero (green monkey kidney, Soroka University Medical Center, Beer-Sheva, Israel) cells were propagated in DMEM (Bio-Lab Ltd., Jerusalem, Israel) supplemented with 10% fetal calf serum (FCS) (Bio-Lab Ltd., Jerusalem, Israel). All cell lines were incubated at 37 °C in an atmosphere of 5% CO_2_.

The HSV-1 KOS strain and acyclovir-resistant strains of HSV-1 and VZV were propagated in Vero cells at the initial multiplicity of infection (m.o.i.) of 0.01 plaque-forming units per cell (pfu/cell). A total of 72 to 96 h post infection, when all cells manifested cytopathic effects, growth medium and host cells were collected and subjected to freeze and thaw procedures three times. The supernatant was centrifuged at 1000 rpm to sediment cell debris, aliquoted and titrated by the plaque assay procedure as previously described [25]. Titrated aliquots were stored at −80 °C.

Equal numbers of cells in 100 μL of an appropriate medium with or without the tested compounds were seeded in 96-well plates. Stock solutions of each chemical were added to obtain the desired concentration. The concentration of phytochemicals was 2 µM. The initial concentration of the extract was 2.5 mg/mL and, after dilution, 0.1 mg/mL. Streptomycin was used as a positive control at the concentration of 2 µM. Plates were incubated at 37 °C for up to 5 days in the atmosphere of 5% CO_2_. The toxicity test was performed either by cell counting once every 24 h or by the WST method on the final day according to the protocol of the manufacturer [26].

Quercetin 3-*O*-rutinoside, quercetin 3-*O*-arabinoside, himachala-2,4-diene, diosmin and hesperidin were purchased from S.L. Moran Ltd (Jerusalem, Israel), and isovaleric aldehyde, 2,4-hexadienal, 2-hexenal, 1-octen-3-ol, α-cubebene, β-bourbonene, n-octanal and α-terpinene were obtained from VK Chemical Services (Jerusalem, Israel). The abovementioned compounds were used as standards and for biological assays.

### 2.6. Antiviral Activity

Host cells were propagated in 6-well plates. Prior to the experiment, the number of cells per well was estimated by cell counting. Four hours before infection, growth media (DMEM (Bio Lab., Ashkelon, Israel)) supplemented with 10% fetal calf serum (FCS) (Bio Lab., Ashkelon, Israel) were replaced by 2.5 mL of fresh media supplemented with the required concentrations of the tested chemicals to allow the accumulation of chemicals inside the cells. After four hours of incubation, the media volumes were reduced to 0.5 mL. Then, the calculated volume of titrated virus stocks was added to the cells. After 2 h of incubation with viruses, media were replaced again with 1.5 mL of fresh media supplemented with the required concentration of tested chemicals. Cells were inspected every 24 h. The experiment was terminated once 100% of infected cells manifested cytopathic effects in the control group (no chemicals added). Viral progenies were collected by subjecting the supernatant and treated cells to three freeze and thaw cycles followed by centrifugation at 1000 rpm to sediment cell debris. The infectious viral yields of HSV-1 and VZV were determined by titration in Vero cells. Toxicity was determined as described previously [23].

### 2.7. Statistical Analysis

At least three independent experiments performed in duplicate were analyzed by Statistica version 13.6 for Windows software (StatSoft, Inc., Tulsa, OK, USA) using Student’s *t*-test. The difference between the results was considered significant when the *p*-value was less than 0.001. Quantitative results are presented as the mean ± standard error.

## 3. Results and Discussion

### 3.1. Identification of Plant Extract Components

**Figure 1:**
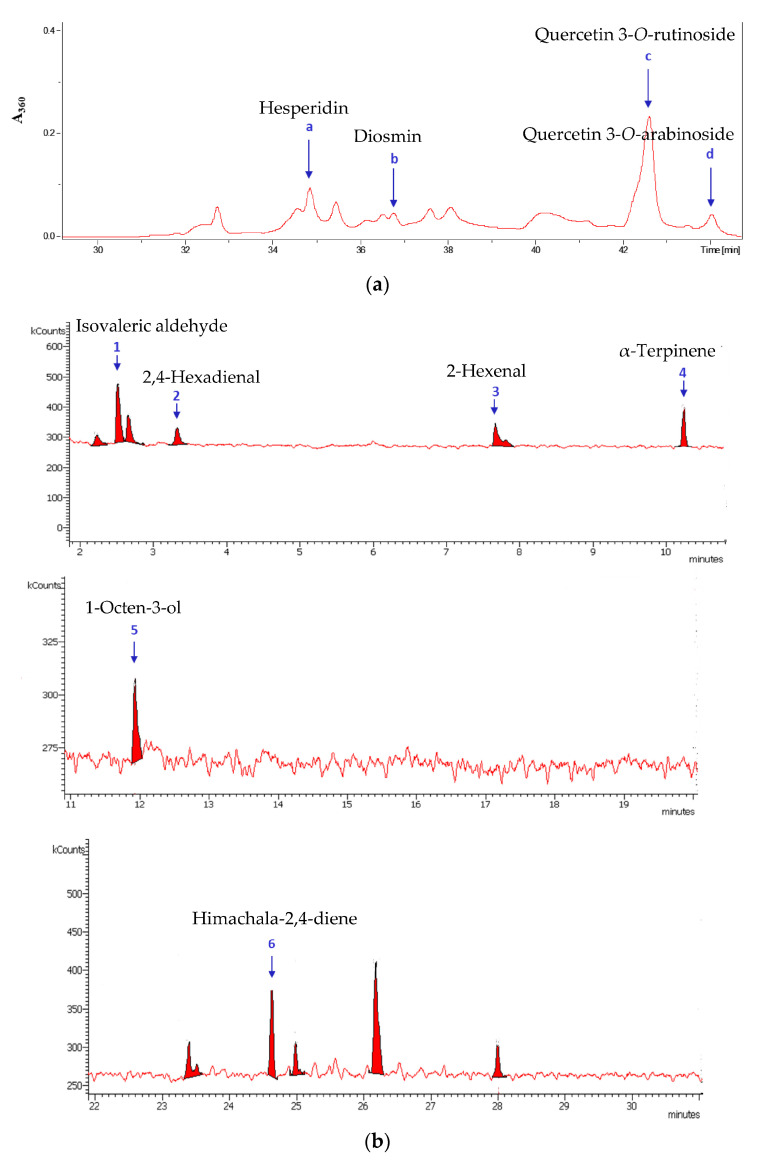

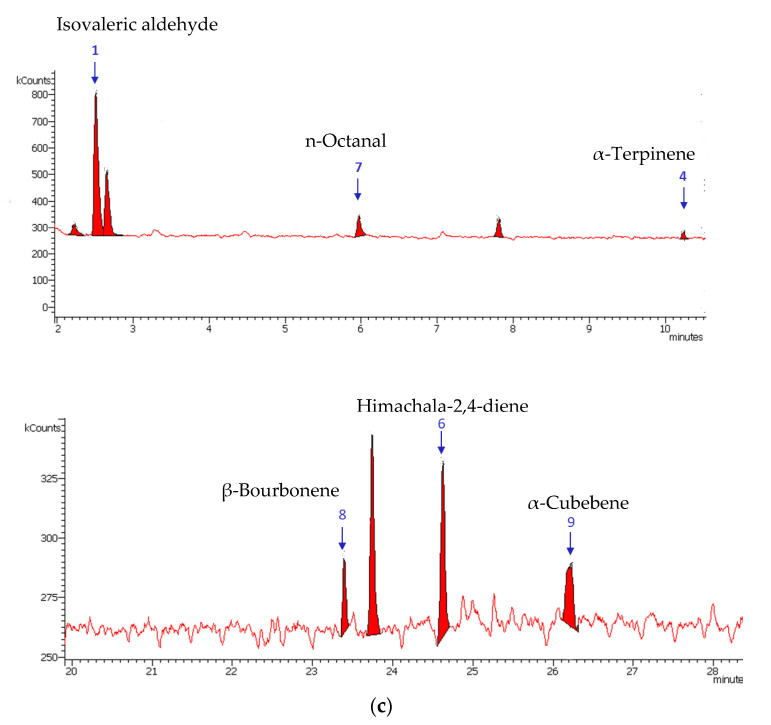
HPLC-MS (**a**) and GC-MS (**b**,**c**) chromatograms of extracts from leaves (**a** and **b**) and flowers (**c**) of *P. viscosa*. The peaks are numbered in the order of compounds listed in Table 1 and Table 2. GC chromatograms are presented in several patterns of ca. 10 min in appropriate signal scales. The GC chromatogram of flowers shows patterns of 0–10 min and 20–30 min since at 10–20 min, there were no identified peaks.

The identified phytochemicals from the leaves and flowers of *P. viscosa* are listed in Table 1 and Table 2. A bioassay-guided fractionation of the ethanolic extract of *P. viscosa* demonstrated that the most active fraction was 80% ethanol. This fraction was used for the identification of nonvolatile components. The GC-MS and HPLC-MS chromatograms are presented in Figure 1. Nonvolatile components were found mostly in leaves (Figure 1a), and volatile components were found in both leaves and flowers (Figure 1b,c; Table 1 and Table 2). Chromatograms also contain multiple peaks that were not identified, but we took into consideration only the peaks whose identification probability was more than 80%. The identification probability was calculated by comparing phytochemicals with standards and compounds in the NIST library. The structures of nonvolatile components were additionally proven by comparison with corresponding commercial standards, thus increasing the probability of their identification to more than 95%. For further antibacterial and antiviral experiments, commercial standards of all the identified components were used.

Figure 1 contains additional information regarding the identified compounds. The identified compounds were purchased and used for further antibacterial and antiviral experiments.

### 3.2. Antimicrobial Properties of Phytochemicals from P. viscosa

Earlier, it was reported that the extract of *P. viscosa* and some phytochemicals were active against *Escherichia coli*, *Staphylococcus aureus* and *Pseudomonas aeruginosa* [4]. In the present research, the plant extract and its individual components were studied in vitro against plant pathogens *Agrobacterium tumefaciens*, *Xylella fastidiosa* and *Pseudomonas syringae*, drug-resistant microorganisms *Escherichia coli*, *Klebsiella pneumoniae*, *Acinetobacter baumannii*, *Serratia marcescens* and *Salmonella enteritidis* and biofilm formation by *Escherichia coli* and *Salmonella enteritidis*.

**Figure 2:**
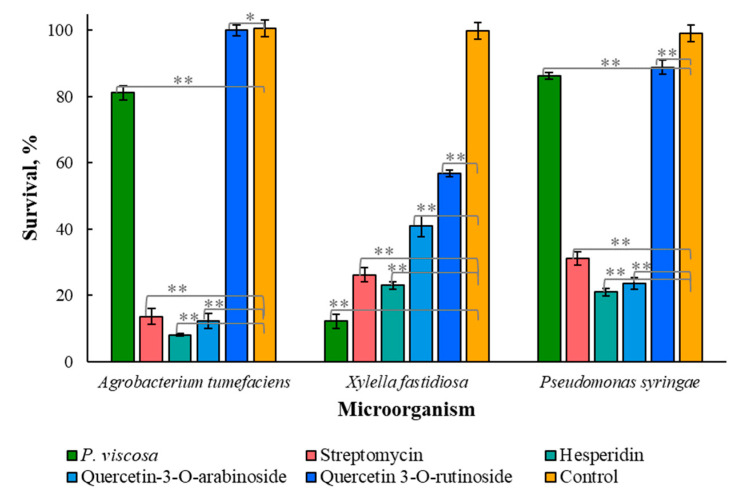
Effect of on eradication of *Agrobacterium tumefaciens*, *Xylella fastidiosa* and *Pseudomonas syringae*. The phytochemicals and streptomycin were at a concentration of 2 µM. Data from three independent experiments are shown. One asterisk designates data of no significant difference, and two asterisks show data of statistically proven difference (*p* < 0.001).

The obtained results (Figure 2) demonstrated that only quercetin 3-*O*-arabinoside and hesperidin were significantly effective against all tested plant pathogens (*p*-value < 0.001), and the other compounds, mentioned in Table 1 and Table 2, did not demonstrate any activity. The phytochemicals and streptomycin, as a positive control, were applied at a concentration of 2 µM. Quercetin 3-*O*-rutinoside and hesperidin caused a significant decrease in the bacterial concentration of *Agrobacterium tumefaciens*, *Xylella fastidiosa* and *Pseudomonas syringae* (*p* < 0.001).

*X. fastidiosa* was sensitive to all phytochemicals tested, including *P. viscosa* crude extract, which killed 88% of the bacteria (Figure 2). At the same time, the effect of the extract on *A. tumefaciens* and *P. syringa* was low when more than 80% of bacteria survived after the treatment. Additionally, both microorganisms were almost insensitive to quercetin 3-*O*-rutinoside. Hesperidin and quercetin 3-*O*-arabinoside were effective against all three tested plant pathogens, with 77–92% microbial kill by hesperidin and 59–88% by quercetin 3-*O*-arabinoside (Figure 2).

To the best of our knowledge, plant extracts and their phytochemicals were never tested against *Agrobacterium tumefaciens*, *Xylella fastidiosa* and *Pseudomonas syringae* examined here.

**Figure 3:**
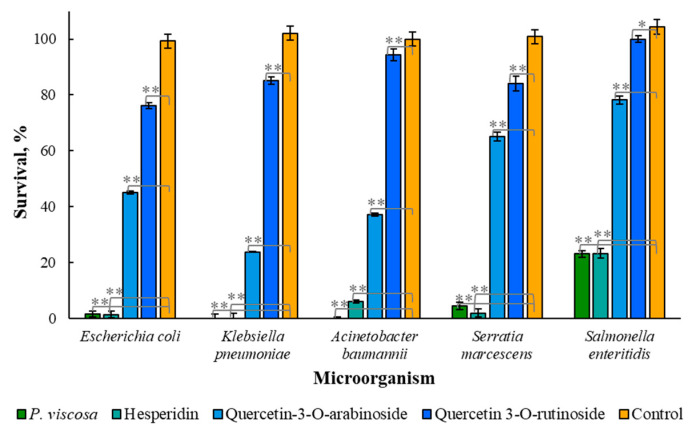
Effect of *P. viscosa* crude extract and its phytochemicals on the eradication of drug-resistant microorganisms (*Escherichia coli*, *Klebsiella pneumoniae*, *Acinetobacter baumannii*, *Serratia marcescens* and *Salmonella enteritidis*). The phytochemicals were applied at a concentration of 2 µM. Data from three independent experiments are shown. One asterisk designates data of no significant difference, and two asterisks show data of statistically proven difference (*p* < 0.001).

The sensitivity of drug-resistant microorganisms *Escherichia coli*, *Klebsiella pneumoniae*, *Acinetobacter baumannii*, *Serratia marcescens* and *Salmonella enteritidis* to the extract and its components was tested at the same concentrations that were used in a previous experiment (Figure 2). Figure 3 demonstrates that quercetin 3-*O*-rutinoside inhibited drug-resistant microorganisms worse than quercetin 3-*O*-arabinoside and hesperidin; hesperidin was the most effective.

As shown in Figure 3, hesperidin and the extract of *P. viscosa* almost completely destroyed all tested bacteria (*E. coli*, *K. pneumoniae*, *A. baumannii* and *S. marcescens*), except for *S. enteritidis*, the destruction of which in both cases was approximately 77%. The antibacterial activity of quercetin 3-*O*-arabinoside was lower; however, it also led to the eradication of 22% (*S. enteritidis*) to 76% (*K. pneumoniae*) of microbial cells. Quercetin 3-*O*-rutinoside showed the lowest antimicrobial activity, reducing the number of live bacteria *E. coli*, *K. pneumoniae* and *S. marcescens* by 15–24%, and had almost no effect on the survival of *A. baumannii* and *S. enteritidis*.

Figure 2 and Figure 3 demonstrate that hesperidin and quercetin 3-*O*-arabinoside were more active than the crude extract since the concentration of each component in extract is lower than when used in our experiments. It is still unknown whether the combined effects of various chemical components of the extract are additive, synergistic or antagonistic.

**Figure 4:**
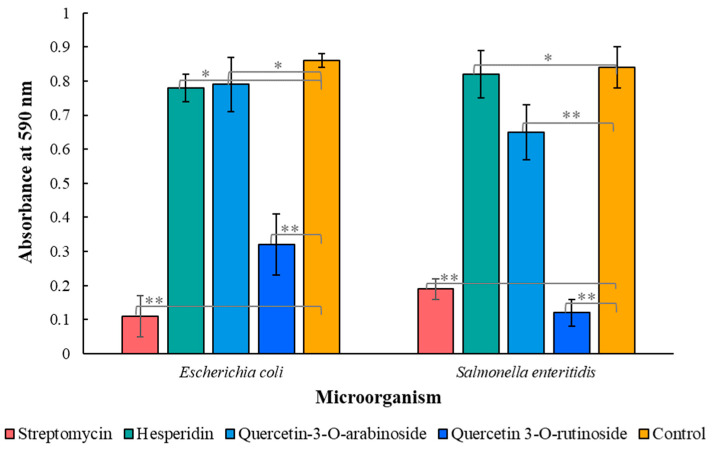
Effect of quercetin 3-*O*-rutinoside, quercetin 3-*O*-arabinoside and hesperidin on biofilm formation. The results are presented as the mean ± SE of the absorbance at 590 nm. Phytochemicals and streptomycin were used at a concentration of 2 µM. Data from three independent experiments are shown. One asterisk designates data of no significant difference, and two asterisks show data of statistically proven difference (*p* < 0.001).

The effect of all identified compounds on biofilm formation was investigated colorimetrically by crystal violet staining. Only quercetin 3-*O*-rutinoside, quercetin 3-*O*-arabinoside and hesperidin inhibited biofilm formation. In experiments with *Escherichia coli*, the effect of the abovementioned compounds was significant (*p* < 0.001) (Figure 4), while streptomycin (positive control) had a weak effect on biofilm formation. A significant decrease in biofilm formation was also observed when *Salmonella enteritidis* bacteria were treated with quercetin 3-*O*-arabinoside and hesperidin (*p* < 0.001) (Figure 4).

Figure 4 shows that hesperidin and quercetin 3-*O*-arabinoside were more active against both microorganisms than streptomycin and quercetin 3-*O*-rutinoside, significantly reducing biofilm formation. The effect of quercetin 3-*O*-rutinoside on biofilm formation for both microorganisms was weaker (Figure 4).

Some publications [27,28] focused on antimicrobial properties of natural crude extracts in which hesperidin is present, but its antimicrobial effect was not tested. Our results showed that hesperidin has significant antimicrobial activity against various microorganisms, including drug-resistant microorganisms (Figure 2 and Figure 3), and it also has the ability to inhibit biofilm formation (Figure 4). Although quercetin 3-*O*-rutinoside and quercetin 3-*O*-arabinoside were mentioned as antimicrobial compounds [6], to the best of our knowledge, their action against drug-resistant microorganisms and antiquorum-sensing properties have not been reported thus far.

All active compounds are flavonoids. The antimicrobial activity of flavonoids relates to several mechanisms, including the inhibition of nucleic acid synthesis via interference in bacterial type II topoisomerases, cytoplasmic membrane function and cell envelope synthesis [6].

### 3.3. Toxicity of Phytochemicals from P. viscosa against Eukaryotic Cells

The first step of antiviral research is the estimation of the toxicity of the tested compounds, and the results of this investigation are demonstrated for Vero (green monkey kidney) cells (Table 3).

Table 3 demonstrates that the extract of *P. viscosa* was not toxic up to a concentration of 500 µg/mL. No cytotoxicity was observed at concentrations below 1000 µg/mL for quercetin 3-*O*-rutinoside. Diosmin, hesperidin and quercetin 3-*O*-arabinoside showed light toxicity at concentrations above 100 µg/mL.

### 3.4. Antiviral Activity of Phytochemicals from P. viscosa

Furthermore, the antiviral activity of the investigated phytochemicals of *P. viscosa*, compared to the traditionally used antiherpetic agent ACV, was tested against varicella zoster virus (VZV) and herpes simplex virus 1 (HSV-1), not ACV-resistant and drug-resistant mutant strains. All the phytochemicals and ACV were applied at the same concentration.

Table 4 shows that diosmin, hesperidin and quercetin 3-*O*-arabinoside significantly inhibited Varicella zoster virus (VZV) (*p* < 0.001). Quercetin 3-*O*-rutinoside and quercetin 3-*O*-arabinoside were effective against SV-1 (Table 4).

As shown in Table 4, diosmin and hesperidin completely destroyed VZV and had no effect on HSV-1. Quercetin 3-*O*-arabinoside was effective against VZV and inhibited HSV-1 viruses by 64%, including the mutant strain. Quercetin 3-*O*-rutinoside was the least active, affecting only the wild strain HSV-1 by almost 40%.

Previous studies also reported differential antiviral activity of plant extracts on HSV-1 and VZV [17,18,23,25,29,30]. *P. viscosa* was never mentioned as an antiviral medicinal plant.

As regards individual components of the *P. viscosa* extract, the antiherpetic properties of quercetin 3-*O*-rutinoside are known [17], but quercetin 3-*O*-arabinoside was never reported as an active compound against HSV-1 and VZV. Diosmin and hesperidin are also demonstrated here for the first time as active compounds against VZV (Table 4).

The elucidation of the mechanisms of action of the tested compounds requires further investigation. The antimicrobial and antiherpetic properties of the identified compounds could be explained, among others, by the antioxidant activity of flavonoids and scavenging capacities [6,18]. It was also shown that some flavonoids are able to interfere with the events occurring between the 3rd and the 9th hour of the HSV-1 replication cycle, which includes transcription and translation of viral proteins [18].

## 4. Conclusions

Quercetin 3-O-rutinoside and hesperidin caused a significant decrease in the bacterial concentration of *Agrobacterium tumefaciens*, *Xylella fastidiosa* and *Pseudomonas syringae*. The growth of drug-resistant microorganisms (*Escherichia coli*, *Klebsiella pneumoniae*, *Acinetobacter baumannii*, *Serratia marcescens* and *Salmonella enteritidis*) was inhibited by quercetin 3-*O*-rutinoside, quercetin 3-*O*-arabinoside and hesperidin. In addition, these compounds demonstrated antiquorum-sensing properties. Diosmin, hesperidin and quercetin 3-*O*-arabinoside significantly inhibited VZV. Quercetin 3-*O*-rutinoside and quercetin 3-*O*-arabinoside were effective against HSV-1 including mutant strains.

The high efficacy and low toxicity of *P. viscosa* extract and its individual components suggest that this plant can serve as a potential natural source for the production of antiviral and antimicrobial compounds. The molecular mechanisms of action of the tested compounds still need to be investigated. In further studies, it will be interesting to investigate the combined (additive, synergistic or antagonistic) effects of multiple chemical components of the extract, for example, a possible synergistic effect of the mixture of quercetin 3-*O*-rutinoside, quercetin 3-*O*-arabinoside, diosmin and hesperidin on biofilm formation. The identified flavonoids are photosensitizers, which allows us to investigate this aspect in the future. Our results also show a perspective to create new drugs and food preservatives based on the identified compounds.

## Figures and Tables

**Table 1:** Phytochemicals extracted from *P. viscosa* and identified by HPLC, LC-ESI-MS and MALDI-TOF-MS.

No	Compound	Molecular Formula	Retention Time, min	Probability of Identification (%)	Origin
a	Hesperidin	C_28_H_34_O_15_	34.85	95.6	Leaves and Flowers
b	Diosmin	C_28_H_32_O_15_	36.76	98.7	Leaves
c	Quercetin 3-*O*-rutinoside	C_27_H_30_O_16_	42.67	96.4	Leaves
d	Quercetin 3-*O*-arabinoside	C_20_H_18_O_11_	44.06	95.9	Leaves

**Table 2:** Phytochemicals extracted from *P. viscosa* and identified by GC-MS.

No	Compound	Molecular Formula	Retention Time, min	Probability of Identification (%)	Origin
1	Isovaleric aldehyde	C_5_H_10_O	2.50	84	Leaves
2	2,4-Hexadienal	C_6_H_8_O	3.33	83.5	Leaves
3	2-Hexenal	C_6_H_10_O	7.66	87.2	Leaves
4	α-Terpinene	C_10_H_16_	10.24	86.5	Leaves and Flowers
5	1-Octen-3-ol	C_8_H_16_O	11.92	87.3	Leaves
6	Himachala-2,4-diene	C_15_H_24_	24.63	85.3	Leaves and Flowers
7	n-Octanal	C_8_H_16_O	5.97	87.3	Flowers
8	β-Bourbonene	C_15_H_24_	23.74	89.2	Flowers
9	α-Cubebene	C_15_H_24_	26.21	87.3	Leaves and Flowers

**Table 3:** Toxicity test of the extract and phytochemicals against eukaryotic cells.

Extract or Compound	Concentration (µg/mL)	Cell Count (% of Control)	Viability (% of Control)
*P. viscosa*	1	101.5 ± 1.4	102.8 ± 2.5
	10	100.7 ± 1.4	100.5 ± 0.9
	100	99.0 ± 1.9	100.9 ± 2.3
	500	100.9 ± 1.9	99.9 ± 4.1
Diosmin	1	101.9 ± 1.6	101.6 ± 1.8
	10	101.2 ± 1.3	102.1 ± 2.2
	100	100.1 ± 1.1	100.7 ± 1.3
	200	72.2 ± 3.1	70.9 ± 2.6
Hesperidin	1	101.5 ± 1.4	100.5 ± 1.6
	10	98.9 ± 1.8	99.2 ± 1.5
	50	87.1 ± 3.1	88.0 ± 2.2
	100	75.3 ± 3.1	74.8 ± 2.5
	200	71.4 ± 1.1	71.9 ± 1.6
Quercetin 3-*O*-arabinoside	1	102.2 ± 1.5	102.7 ± 1.8
	10	97.3 ± 1.2	97.6 ± 1.4
	50	94.2 ± 2.5	94.7 ± 2.3
	100	89.7 ± 1.2	89.2 ± 1.4
	200	84.8 ± 1.1	84.1 ± 2.0
Quercetin 3-*O*-rutinoside	1	103.2 ± 1.9	103.6 ± 1.2
	10	102.2 ± 1.2	101.9 ± 1.7
	100	100.4 ± 1.5	100.7 ± 1.7
	200	100.7 ± 1.3	100.4 ± 2.0
	1000	100.1 ± 2.2	100.2 ± 1.8

**Table 4:** Antiviral properties of phytochemicals of *P. viscosa*.

Compound	Fraction of Plaque Forming Units		
	HSV-1	HSV-1, Mutant	VZV
Untreated control	1	1	1
Diosmin	1	1	0
Hesperidin	1	1	0
Quercetin 3-*O*-arabinoside	0.35 ± 0.05	0.36 ± 0.03	0.11 ± 0.07
Quercetin 3-*O*-rutinoside	0.61 ± 0.08	1	1
Acyclovir	0.12 ± 0.02	1	0

## Data Availability

The data are available in this publication.
